# Prediction of stroke volume response to fluid bolus in 100 children

**DOI:** 10.1186/cc12145

**Published:** 2013-03-19

**Authors:** R Saxena, A Durward, I Murdoch, S Tibby

**Affiliations:** 1 Guy's & St Thomas' NHS Trust, London, UK

## Introduction

Fluid overload is associated with poor outcome in the critically ill. Thus, an accurate predictor of a positive haemodynamic response (increase in stroke volume) to fluid challenge is vital.

## Methods

We studied the predictive value (positive response defined as change in stroke volume >15% after 10 ml/kg fluid bolus) of a range of haemodynamic variables: static (CVP, active circulating volume, central blood volume, total end diastolic volume), dynamic (systolic pressure variation, stroke volume variation) and contactility (dp/dt), in a group of 100 ventilated children (median weight 10 kg). Variables were measured using transpulmonary ultrasound dilution and PRAM (an arterial pulse contour method).

## Results

We performed 168 paired measurements (pre-fluid and post-fluid challenge), with a SV response rate of 45%. Overall predictive values were poor, but slightly better for static versus dynamic variables (Table 1). When SV response was analysed as a continuous variable, the two predictive multivariable variables were change in TEDVI and baseline dp/dt (*r*^2 ^= 0.30, both *P <*0.001).

**Table 1:** ROC areas for haemodynamic variables

	AUC	95% CI	
CVP	0.50	0.41	0.59
ACVI	0.62	0.54	0.71
CBVI	0.59	0.50	0.68
TEDVI	0.65	0.56	0.73
SPV	0.59	0.50	0.69
SVV	0.48	0.39	0.58
dp/dt	0.59	0.50	0.68

## Conclusion

The predictive ability for typical static and dynamic haemodynamic variables, when taken in isolation, is poor. However, improved prediction is seen when baseline contractility is taken into account.

